# Autophagy prevents hippocampal α-synuclein oligomerization and early cognitive dysfunction after anesthesia/surgery in aged rats

**DOI:** 10.18632/aging.103074

**Published:** 2020-04-26

**Authors:** Ning Yang, Zhengqian Li, Dengyang Han, Xinning Mi, Miao Tian, Taotao Liu, Yue Li, Jindan He, Chongshen Kuang, Yiyun Cao, Lunxu Li, Cheng Ni, John Q. Wang, Xiangyang Guo

**Affiliations:** 1 Department of Anesthesiology, Peking University Third Hospital, Beijing 100191, China; 2 Chinese Traditional and Herbal Drugs Editorial Office, Tianjin Institute of Pharmaceutical Research, Tianjin 300193, China; 3 Department of Anesthesiology, Shanghai Sixth People’s Hospital East Affiliated with Shanghai University of Medicine and Health Sciences, Shanghai 200233, China; 4 Department of Anesthesiology, Peking University International Hospital, Beijing 102200, China; 5 Department of Anesthesiology, University of Missouri Kansas City, School of Medicine, Kansas, MO 64110, USA

**Keywords:** autophagy, postoperative cognitive dysfunction (POCD), α-synuclein, oligomerization, hippocampus

## Abstract

Stress-induced α-synuclein aggregation, especially the most toxic species (oligomers), may precede synaptic and cognitive dysfunction. Under pathological conditions, α-synuclein is degraded primarily through the autophagic/lysosomal pathway. We assessed the involvement of autophagy in α-synuclein aggregation and cognitive impairment following general anesthesia and surgical stress. Autophagy was found to be suppressed in the aged rat hippocampus after either 4-h propofol anesthesia alone or 2-h propofol anesthesia during a laparotomy surgery. This inhibition of autophagy was accompanied by profound α-synuclein oligomer aggregation and neurotransmitter imbalances in the hippocampus, along with hippocampus-dependent cognitive deficits. These events were not observed 18 weeks after propofol exposure with or without surgical stress. The pharmacological induction of autophagy using rapamycin markedly suppressed α-synuclein oligomerization, restored neurotransmitter equilibrium, and improved cognitive behavior after prolonged anesthesia or anesthesia combined with surgery. Thus, both prolonged propofol anesthesia alone and propofol anesthesia during surgery impaired autophagy, which may have induced abnormal hippocampal α-synuclein aggregation and neurobehavioral deficits in aged rats. These findings suggest that the activation of autophagy and the clearance of pathological α-synuclein oligomers may be novel strategies to ameliorate the common occurrence of postoperative cognitive dysfunction.

## INTRODUCTION

Postoperative cognitive dysfunction (POCD) is a decline in a patient’s cognitive function after a surgery [1], and is most prevalent in the elderly [2]. In a previous study, approximately 12% of older adults exhibited cognitive dysfunction for at least three months following surgery [2]. POCD has been linked to premature departure from the workforce, increased disability and early mortality [1, 3]. The etiology of POCD remains unclear, and there are few effective clinical interventions to prevent this disorder.

Autophagy is a natural, lysosome-induced process in which damaged organelles and unnecessary long-lived proteins are degraded and recycled [4]. Although little is known about the relationship between autophagy and POCD, impaired autophagy following sevoflurane anesthesia was found to induce cognitive dysfunction in aged rats [5]. We previously reported that 1.5% isoflurane exposure reduced both spatial cognitive function and hippocampal phagophore formation in aged rats [6]. In another study, we found that propofol anesthesia alone for 4 h significantly hampered cognitive performance by inhibiting hippocampal autophagy [7]. Nevertheless, propofol-induced POCD has been poorly characterized, and it is not known whether impaired autophagy also contributes to POCD when propofol anesthesia is combined with surgery.

Anesthesia and surgery can promote POCD by inducing synaptic dysfunction [8, 9]. Synaptic dysfunction is associated with the abnormal expression of proteins needed for synaptic function [1, 10]. α-Synuclein is a 140-amino-acid protein found primarily in presynaptic terminals. At presynaptic boutons, α-synuclein may help to maintain neurotransmitter homeostasis by regulating synaptic vesicle fusion, clustering and trafficking between the reserve and ready-releasable pools, as well as by binding to neurotransmitter membrane transporters [11, 12]. α-Synuclein is a non-amyloid-β component of the amyloid plaques in Alzheimer’s disease patients’ brains [13]. In addition, α-synuclein aggregates have been found in the brains of patients with various neurodegenerative diseases, including Parkinson’s disease, Parkinson’s disease dementia, dementia with Lewy bodies, multiple system atrophy and the Lewy body variant of Alzheimer’s disease [12]. Metal ions, oxidative stress, post-translational modifications and ubiquitin-proteasome system activity have been implicated in α-synuclein misfolding and oligomerization [14].

In a previous study by Ren et al., mice that had undergone anesthesia and surgery exhibited elevated total α-synuclein levels in the cortex after 12 h and loss of attention after 24 h [15]. In addition, significant aggregation of phosphorylated α-synuclein was observed in the myenteric plexus in patients who experienced postoperative delirium after a gastrectomy due to stomach cancer [16]. Considered along with the finding that pathologic α-synuclein can spread from the gastrointestinal nervous system to the brain [17], these data imply that α-synuclein and/or its oligomers may contribute to the development of POCD. In this study, we investigated whether impaired autophagy promoted α-synuclein aggregation and POCD following propofol anesthesia with or without laparotomy surgery in aged rats.

## RESULTS

### Propofol anesthesia alone caused neurobehavioral deficits

**Figure 1:**
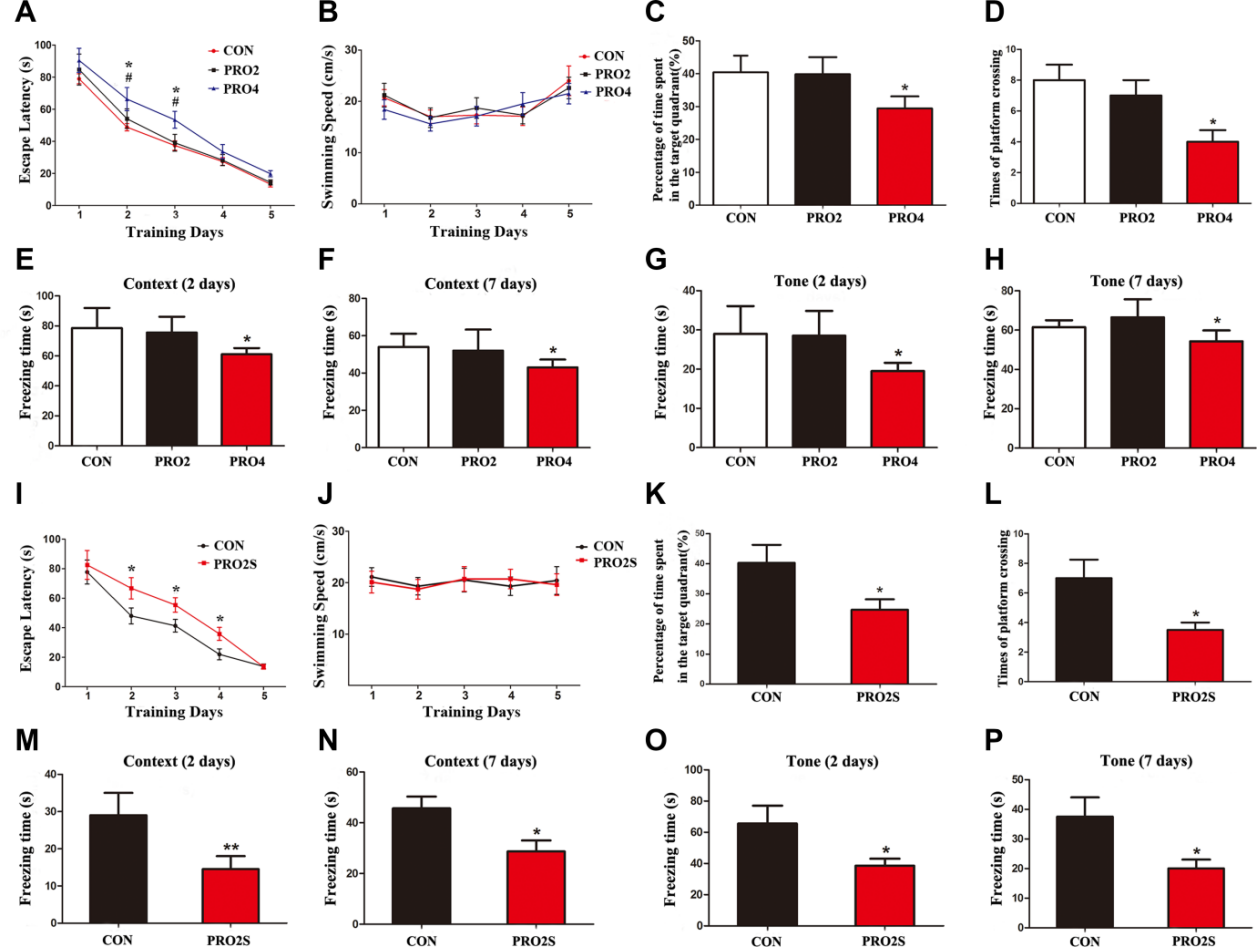
**The effects of propofol anesthesia with or without surgery on learning and memory behaviors in aged rats.** (**A**) Propofol alone for 4 h but not 2 h increased the escape latency in the MWM test. (**B**) Propofol alone did not alter the swimming speed. (**C**) Propofol alone for 4 h but not 2 h reduced the percentage of time spent in the target quadrant. (**D**) Propofol alone for 4 h but not 2 h reduced the number of platform crossings. (**E**, **F**) Propofol alone for 4 h reduced the freezing time in the context test of the FCT both 2 and 7 days after anesthesia. (**G**, **H**) Propofol alone for 4 h reduced the freezing time in the tone test of the FCT both 2 and 7 days after anesthesia. (**I**–**L**) Propofol anesthesia (2 h) and surgery increased the escape latency (**I**), had no effect on the swimming speed (**J**), reduced the target quadrant dwelling time (**K**) and reduced the number of platform crossings (**L**) in the MWM test. (**M**–**P**) Propofol anesthesia (2 h) and surgery reduced the freezing time in the context (**M**, **N**) and tone (**O**, **P**) tests of the FCT 2 and 7 days after anesthesia. *p < 0.05 and **p < 0.01 versus control rats. Data are expressed as the mean ± SEM (n = 10 per group). CON: the control group, PRO2: the 2-h propofol group, PRO4: the 4-h propofol group, PRO2S: the 2-h propofol anesthesia + surgery group.

We first examined the impact of different durations of propofol anesthesia (2 h versus 4 h) on learning and memory behaviors in aged rats. Rats that had been anesthetized with propofol for 2 h did not differ significantly from control rats in their performance of the Morris water maze (MWM) test (*p* > 0.05; Figure 1A–1D). On the other hand, rats that had been anesthetized with propofol for 4 h exhibited longer escape latencies on test days 2 and 3 (*p* < 0.05; Figure 1A), shorter exploration time (*p* < 0.05; Figure 1C) and fewer platform crossings than control rats (*p* < 0.05; Figure 1D). The swimming speeds were similar among the three groups (*p* > 0.05; Figure 1B). In a fear conditioning test (FCT), the freezing time (a measure of fear memory) did not differ significantly between the control rats and the 2-h propofol-treated rats on day 2 or 7 after anesthesia (*p* > 0.05; Figure 1E–1H). In contrast, on post-anesthesia days 2 and 7, the freezing time in both the context and tone tests were significantly shorter in 4-h propofol-anesthetized rats than in control rats (*p* < 0.05; Figure 1E–1H). These results demonstrate that a longer period of propofol anesthesia (4 h) was more likely than a shorter period of anesthesia (2 h) to impair learning and memory behaviors in aged rats.

### Propofol anesthesia combined with surgery caused neurobehavioral deficits

We next investigated the behavioral effects of propofol anesthesia (2 h) plus surgery (laparotomy) in the two behavioral assays. In the MWM test, rats subjected to anesthesia and surgery displayed greater escape latencies on test days 2, 3 and 4 (all *p* < 0.05; Figure 1I), shorter exploration time (*p* < 0.05; Figure 1K) and fewer platform quadrant crossings (*p* < 0.05; Figure 1L) than control rats. However, the swimming speeds did not differ between the two groups (*p* > 0.05; Figure 1J). In the FCT, the freezing time in the context and tone tests on days 2 and 7 after surgery were significantly shorter in the anesthesia/surgery group than in the control group (*p* < 0.05 or 0.01; Figure 1M–1P). Thus, when combined with surgery, 2-h propofol anesthesia impaired learning and memory behaviors in aged rats.

### Both propofol alone (4 h) and propofol (2 h)/surgery inhibited autophagy and increased α-synuclein oligomerization in the hippocampus

**Figure 2:**
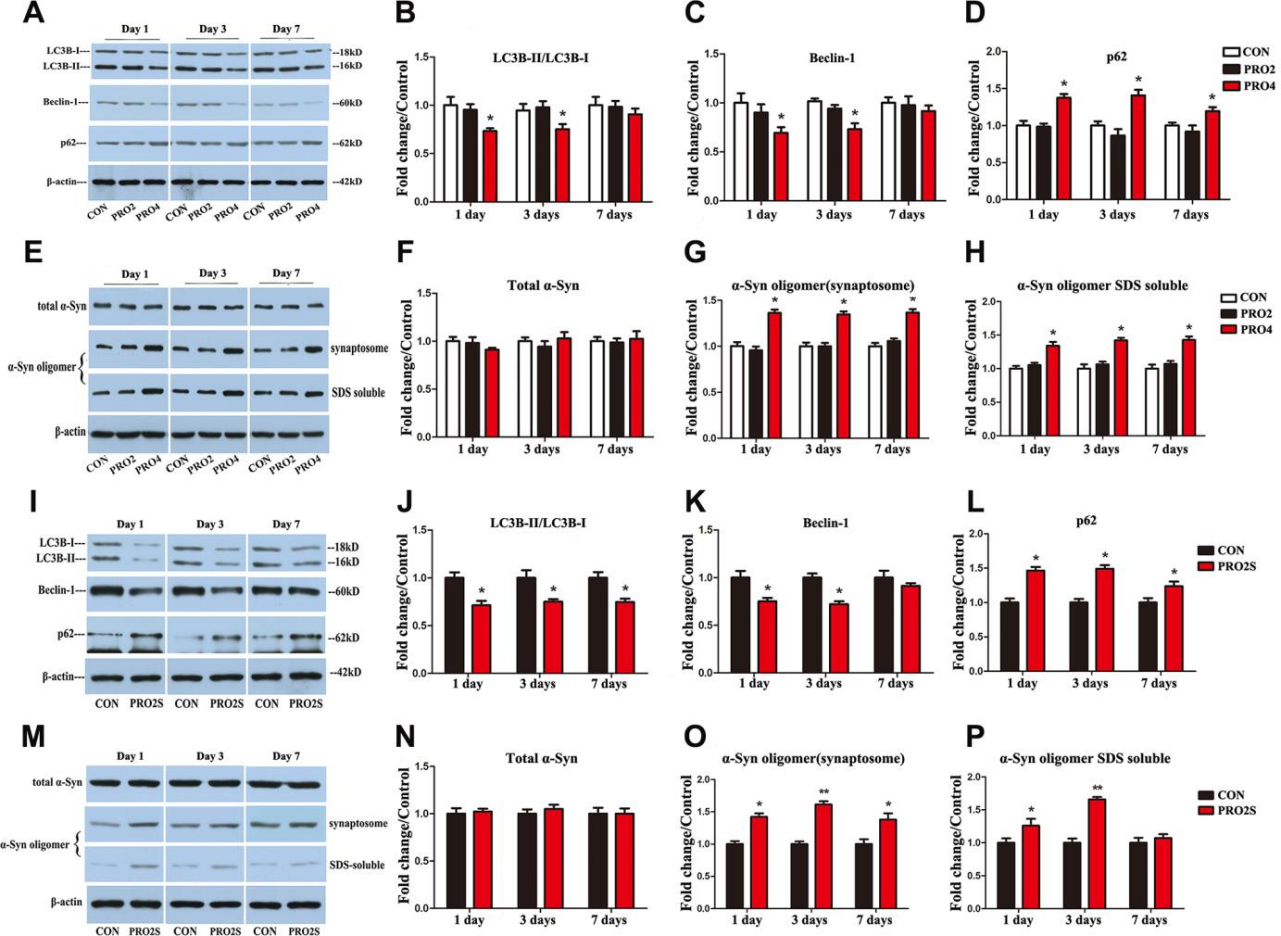
**The effects of propofol anesthesia with or without surgery on autophagy-related protein, total α-synuclein and α-synuclein oligomer levels in aged rats.** (**A**) Representative immunoblots illustrating autophagy-related protein levels in the hippocampus on days 1, 3 and 7 after propofol anesthesia. (**B**) Propofol (4 h) reduced LC3B expression in the hippocampus on days 1 and 3 post-anesthesia. (**C**) Propofol (4 h) reduced Beclin-1 expression in the hippocampus on days 1 and 3 post-anesthesia. (**D**) Propofol (4 h) increased p62 expression in the hippocampus on days 1, 3 and 7 post-anesthesia. (**E**) Representative immunoblots illustrating total α-synuclein (α-Syn) and α-synuclein oligomer levels in the hippocampus on days 1, 3 and 7 after propofol anesthesia. (**F**) Propofol did not alter total α-synuclein expression. (**G**, **H**) Propofol (4 h) elevated α-synuclein oligomer expression in both synaptosomes and SDS-solubilized fractions. (**I**) Representative immunoblots illustrating autophagy-related protein levels in the hippocampus on days 1, 3 and 7 after propofol anesthesia (2 h) and surgery. (**J**) Propofol anesthesia and surgery reduced LC3B expression in the hippocampus. (**K**) Propofol anesthesia and surgery reduced Beclin-1 expression in the hippocampus. (**L**) Propofol anesthesia and surgery increased p62 expression in the hippocampus. (**M**) Representative immunoblots illustrating total α-synuclein and α-synuclein oligomer levels in the hippocampus on days 1, 3 and 7 after propofol anesthesia (2 h) and surgery. (**N**) Propofol anesthesia and surgery did not significantly alter total α-synuclein expression. (**O**) Propofol anesthesia and surgery elevated α-synuclein oligomer levels in hippocampal synaptosomes. (**P**) Propofol anesthesia and surgery elevated α-synuclein oligomer levels in SDS-solubilized hippocampal samples. Values are expressed as the mean ± SEM (n = 6 per group). *p < 0.05 and **p < 0.01 versus control rats. CON: the control group, PRO2: the 2-h propofol group, PRO4: the 4-h propofol group, PRO2S: the 2-h propofol anesthesia + surgery group.

To determine the effects of propofol anesthesia with or without surgery on autophagy, we performed Western blotting to monitor autophagic marker expression (LC3B, Beclin-1 and p62) in the hippocampus. On days 1 and 3 after anesthesia, both LC3B and Beclin-1 protein levels in the hippocampus were lower in the 4-h propofol anesthesia group than in the control group (*p* < 0.05; Figure 2A–2C). In contrast, p62 protein levels in the hippocampus on days 1, 3 and 7 following anesthesia were greater in the 4-h propofol anesthesia group than in the control group (Figure 2A, 2D). Hippocampal α-synuclein oligomer levels in synaptosomal samples and sodium dodecyl sulfate (SDS)-solubilized fractions on days 1, 3 and 7 after anesthesia were also higher in the 4-h propofol anesthesia group than in the control group (Figure 2E, 2G, 2H), while total α-synuclein levels were unaltered by anesthesia (Figure 2E, 2F). However, propofol anesthesia for 2 h had no significant effect on the expression of the three autophagic markers or α-synuclein in the hippocampus. These data indicate that propofol anesthesia for 4 h, but not 2 h, concomitantly suppressed autophagy and stimulated α-synuclein oligomerization in the hippocampus.

While propofol anesthesia for 2 h had no effect on the above parameters, 2-h anesthesia combined with surgery reduced LC3B and Beclin-1 levels and increased p62 levels in the hippocampus on post-surgery days 1, 3 and 7 (*p* < 0.05; Figure 2I–2L). Synaptosomal α-synuclein oligomer levels were also greater in the 2-h propofol anesthesia/surgery-treated rats than in the control rats at the three time points surveyed (Figure 2M, 2O). Similar increases in α-synuclein levels in SDS-solubilized fractions were observed on days 1 and 3 (Figure 2M, 2P), while total α-synuclein expression did not differ significantly between the two groups (*p* > 0.05; Figure 2M, 2N). Thus, 2-h propofol anesthesia combined with surgery inhibited autophagy and promoted α-synuclein oligomerization in the hippocampus.

### Rapamycin mitigated the hippocampal α-synuclein oligomer aggregation, neurotransmitter imbalances and cognitive deficits induced by propofol anesthesia

**Figure 3:**
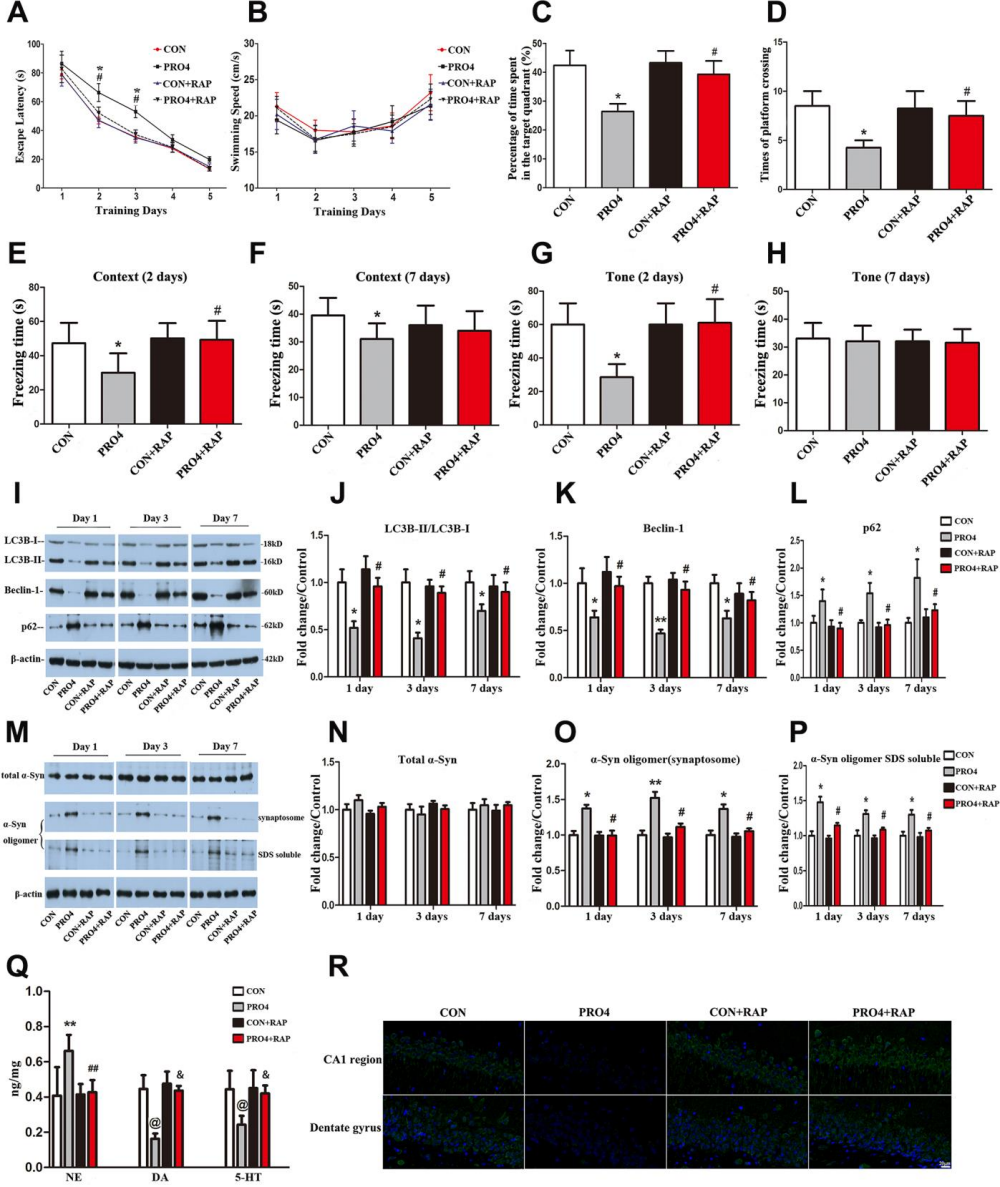
**The effects of an autophagy agonist on propofol-induced changes in behavior, autophagy-related protein levels and α-synuclein levels in the hippocampus in aged rats.** (**A**) Rapamycin (RAP) reversed the propofol anesthesia (4 h)-induced increase in escape latency. (**B**) Propofol anesthesia (4 h) and/or rapamycin did not alter the swimming speed. (**C**, **D**) Rapamycin reversed the propofol anesthesia (4 h)-induced decreases in the target quadrant dwelling time and the number of platform crossings. (**E**, **F**) Rapamycin ameliorated the propofol anesthesia (4 h)-induced decrease in the freezing time in the context test 2 and 7 days after anesthesia. (**G**, **H**) Rapamycin ameliorated the propofol anesthesia (4 h)-induced decrease in the freezing time in the tone test 2 and 7 days after anesthesia. (**I**) Representative immunoblots illustrating the effects of rapamycin on the propofol anesthesia (4 h)-induced changes in hippocampal autophagy-related protein levels. (**J**) Rapamycin reversed the propofol anesthesia (4 h)-induced decrease in LC3B expression in the hippocampus. (**K**) Rapamycin reversed the propofol anesthesia (4 h)-induced decrease in Beclin-1 expression in the hippocampus. (**L**) Rapamycin reversed the propofol anesthesia (4 h)-induced increase in p62 expression in the hippocampus. (**M**) Representative immunoblots illustrating the effects of rapamycin on the propofol anesthesia (4 h)-induced changes in α-synuclein (α-Syn) levels in the hippocampus. (**N**) Rapamycin did not alter total α-synuclein levels. **(O**, **P**) Rapamycin reversed the propofol anesthesia (4 h)-induced increases in α-synuclein oligomer levels in synaptosomes and SDS-solubilized fractions in the hippocampus. (**Q**) Rapamycin reversed the propofol anesthesia (4 h)-induced changes in neurotransmitter levels in the hippocampus. (**R**) Representative confocal staining of LC3B expression in the CA1 and DG regions of the hippocampus in the four groups of rats 7 days after propofol anesthesia. Values are expressed as the mean ± SEM (n = 10 per group for behavioral tests, n = 8 per group for neurotransmitter detection, n = 6 per group for Western blot analysis, n = 4 per group for confocal analysis of LC3B staining). *p < 0.05, **p < 0.01, ^@^*p* < 0.001 PRO4 vs. CON group. #*p* < 0.05, ##*p* < 0.01, ^&^*p* < 0.001 PRO4 + RAP group vs. PRO4 group. CON: the control group, CON + RAP: the control + rapamycin group, PRO4: the 4-h propofol anesthesia group, PRO4 + RAP: the 4-h propofol anesthesia + rapamycin group. Scale bar, 20 μm (**R**).

To determine whether propofol anesthesia induced α-synuclein oligomerization and cognitive dysfunction by inhibiting autophagy, we pretreated the rats with the autophagy agonist rapamycin (10 mg/kg, injected intraperitoneally once daily for five days). On the sixth day, the rats were injected with rapamycin 1 h prior to propofol anesthesia (4 h). We then examined the effects of rapamycin on propofol-induced behavioral deficits and neurochemical changes on different days after anesthesia. Remarkably, in the MWM test, rapamycin reversed the increased escape latencies induced by propofol anesthesia (4 h) on days 2 and 3 after anesthesia (*p* < 0.05; Figure 3A). Rapamycin also reversed the reduced exploration time (*p* < 0.05; Figure 3C) and number of platform crossings (*p* < 0.05; Figure 3D) on post-anesthesia day 6. In the FCT, the freezing time in the context (days 2) and tone (day 2) tests were significantly longer in the anesthesia + rapamycin group than in the anesthesia alone group (*p* < 0.05; Figure 3E–3G). In fact, the freezing time on days 2 and 7 did not differ significantly between control rats and rats treated with anesthesia + rapamycin (*p* > 0.05; Figure 3E–3H). The control and propofol-anesthetized rats did not differ significantly in their performance of the tone test on day 7 (*p* > 0.05; Figure 3H). These behavioral data indicate that enhancing autophagy can prevent propofol from inducing cognitive deficits.

In neurochemical assays with hippocampal samples, we found that rapamycin pretreatment abolished the 4-h propofol-induced decreases in LC3B and Beclin-1 levels and increases in p62 levels on days 1, 3 and 7 after anesthesia (*p* < 0.05; Figure 3I–3L and 3R). The levels of these three autophagic markers in rats treated with propofol (4 h) and rapamycin did not differ from those in control rats treated with or without rapamycin (*p* > 0.05; Figure 3I–3L). These data establish that rapamycin can reverse the propofol-induced inhibition of autophagy.

More interestingly, rapamycin also reversed the propofol-induced oligomerization of α-synuclein. In the presence of rapamycin, propofol anesthesia (4 h) no longer increased the abundance of α-synuclein oligomers in synaptosomal and SDS-solubilized samples from the hippocampus on days 1, 3 and 7 after anesthesia (*p* < 0.05; Figure 3M, 3O, 3P). This indicates that the inhibition of autophagy was likely a prerequisite for the oligomerization of α-synuclein in response to propofol anesthesia.

Propofol (4 h) also seemed to alter local neurotransmitter levels in the hippocampus. Hippocampal norepinephrine levels were upregulated but dopamine and 5-hydroxytryptamine levels were downregulated in propofol-treated rats relative to control rats (Figure 3Q). Rapamycin pretreatment reversed these changes. Rapamycin alone did not alter the basal levels of these neurotransmitters in the hippocampus.

### Changes in α-synuclein oligomerization, neurotransmitter levels and cognitive behavior were no longer evident 18 weeks after propofol anesthesia

We also examined rats 18 weeks after propofol anesthesia. The escape latency, percentage of time spent in the target quadrant and swimming speed did not differ between the propofol anesthesia group and the control group 18 weeks after anesthesia (all *p* > 0.05; Supplementary Figure 1A–1D). α-Synuclein oligomers and autophagy-related proteins in the hippocampus also returned to control levels 18 weeks after anesthesia (all *p* > 0.05; Supplementary Figure 1E–1H and 1J). There were no significant differences in hippocampal neurotransmitter levels between the two groups (all *p* > 0.05; Supplementary Figure 1I).

### Rapamycin reversed the changes in α-synuclein oligomerization, neurotransmitter levels and cognitive behavior induced by propofol anesthesia and surgery

**Figure 4:**
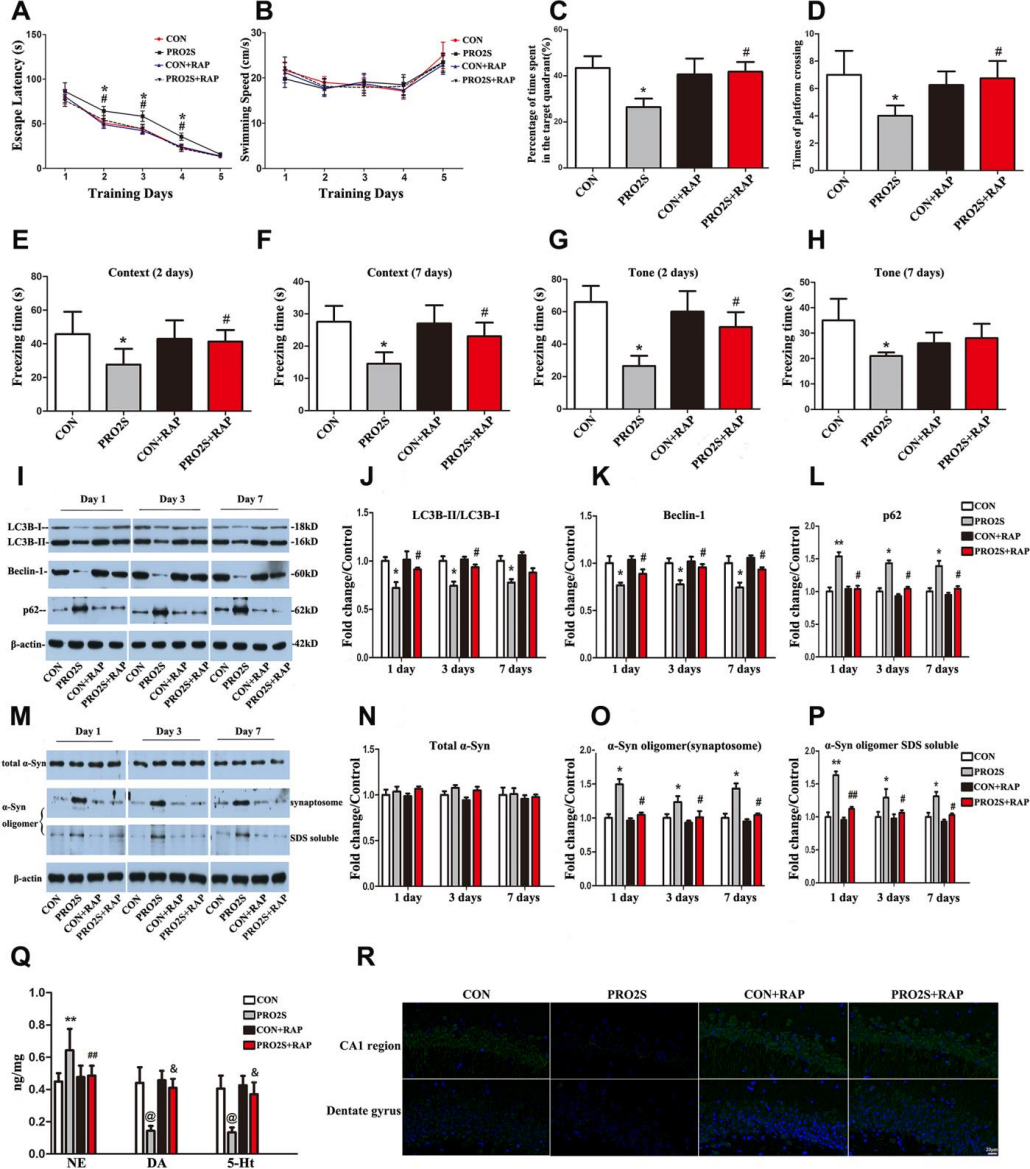
**The effects of an autophagy agonist on changes in behavior, autophagy-related protein levels and α-synuclein levels induced by propofol anesthesia and surgery in aged rats.** (**A**) Rapamycin (RAP) reversed the increased escape latency induced by propofol anesthesia (2 h) and surgery. (**B**) Propofol anesthesia (2 h) and surgery with or without rapamycin did not alter the swimming speed. (**C**, **D**) Rapamycin reversed the reduced target quadrant dwelling time and number of platform crossings induced by propofol anesthesia (2 h) and surgery. (**E**, **F**) Rapamycin ameliorated the reduced freezing time in the context test induced by propofol anesthesia (2 h) and surgery, both 2 and 7 days after surgery. (**G**, **H**) Rapamycin ameliorated the reduced freezing time in the tone test induced by propofol anesthesia (2 h) and surgery on day 2 but not day 7 after surgery. (**I**) Representative immunoblots illustrating the effects of rapamycin on the changes in hippocampal autophagy-related protein levels induced by propofol anesthesia (2 h) and surgery. (**J**) Rapamycin reversed the decrease in LC3B expression in the hippocampus induced by propofol anesthesia (2 h) and surgery. (**K**) Rapamycin reversed the decrease in Beclin-1 expression in the hippocampus induced by propofol anesthesia (2 h) and surgery. (**L**) Rapamycin reversed the increase in p62 expression in the hippocampus induced by propofol anesthesia (2 h) and surgery. (**M**) Representative immunoblots illustrating the effects of rapamycin on the changes in α-synuclein (α-Syn) levels in the hippocampus induced by propofol anesthesia (2 h) and surgery. (**N**) Rapamycin did not alter total α-synuclein expression. (**O**, **P**) Rapamycin reversed the α-synuclein oligomerization in synaptosomes and SDS-solubilized fractions in the hippocampus induced by propofol anesthesia (2 h) and surgery. (**Q**) Rapamycin reversed the changes in neurotransmitter levels in the hippocampus induced by propofol anesthesia (2 h) and surgery. (**R**) Representative confocal staining of LC3B expression in the CA1 and DG regions of the hippocampus in the four groups of rats 7 days after propofol anesthesia. Values are expressed as the mean ± SEM (n = 10 per group for behavioral tests, n = 8 per group for neurotransmitter detection, n = 6 per group for Western blot analysis, n = 4 per group for confocal analysis of LC3B staining). *p < 0.05, **p < 0.01, ^@^*p* < 0.001 PRO2S vs. CON group. #*p* < 0.05, ##*p* < 0.01, ^&^*p* < 0.001 PRO2S + RAP group vs. PRO2S group. CON: the control group, CON + RAP: the control + rapamycin group, PRO2S: the 2-h propofol anesthesia + surgery group, PRO2S + RAP: the 2-h propofol anesthesia + surgery + rapamycin group. Scale bar, 20 μm (**R**).

To determine whether propofol anesthesia combined with surgery induced α-synuclein oligomerization and cognitive dysfunction by inhibiting autophagy, we pretreated rats with the autophagy agonist rapamycin. As expected, propofol anesthesia (2 h) plus surgery increased the escape latencies in the MWM test 2-4 days after anesthesia and surgery (*p* < 0.05; Figure 4A). Rapamycin abolished these changes. Rapamycin also reversed the reduced exploration time (*p* < 0.05; Figure 4C) and number of platform crossings (*p* < 0.05; Figure 4D) in the anesthesia/surgery group. In the FCT, propofol anesthesia (2 h) and surgery reduced the freezing time in the context test on days 2 and 7, while this was not observed in the presence of rapamycin (Figure 4E and 4F). In the tone test, rapamycin reversed the reduced freezing time induced by propofol anesthesia and surgery on day 2, but not on day 7 (*p* < 0.05; Figure 4G and 4H). Thus, the activation of autophagy with rapamycin corrected the abnormal behavioral responses to propofol anesthesia (2 h) and surgery.

In addition, rapamycin significantly impacted the response of autophagy-related proteins to propofol anesthesia and surgery. As shown in Figure 4I, 4J and 4R, after the rats were pretreated with rapamycin, no significant differences in hippocampal LC3B expression were detected between anesthesia/surgery-treated rats and control rats on days 1, 3 and 7 after anesthesia and surgery. Similar results were observed for Beclin-1 and p62 expression in the hippocampus (Figure 4I, 4K and 4L).

Rapamycin also altered α-synuclein oligomer expression in the hippocampus. After the rats were pretreated with rapamycin, the α-synuclein oligomer levels in synaptosomes and SDS-solubilized fractions did not differ significantly between anesthesia/surgery-exposed rats and control rats (*p* > 0.05; Figure 4M, 4O and 4P). These results were consistent on all three test days (1, 3 and 7 days after anesthesia and surgery). Neither anesthesia and surgery nor rapamycin altered total α-synuclein expression (*p* > 0.05; Figure 4M and 4N). These results indicate that the autophagy agonist rapamycin can reverse the autophagy-related protein expression changes and α-synuclein oligomerization induced by anesthesia and surgery.

Surgery and anesthesia altered neurotransmitter levels in the hippocampus. Hippocampal norepinephrine levels were greater, while dopamine and 5-hydroxytryptamine levels were lower in 2-h propofol anesthesia/surgery-treated rats than in control rats (Figure 4Q). Rapamycin abolished these changes in the hippocampus.

### Changes in α-synuclein oligomerization, neurotransmitter levels and cognitive behavior were reversed 18 weeks after surgery plus anesthesia

We also examined rats 18 weeks after propofol anesthesia and surgery. At this longer time point, no differences in escape latency, percentage of time spent in the target quadrant or swimming speed were found among the groups (all *p* > 0.05; Supplementary Figure 2A–2D). Hippocampal levels of autophagy-related proteins and α-synuclein oligomers also returned to the control level at 18 weeks (all *p* > 0.05; Supplementary Figure 2E–2H and 2J). There were no significant differences in hippocampal neurotransmitter levels among the groups (all *p* > 0.05; Supplementary Figure 2I).

### Blood gas analysis

There were no significant differences in the blood concentrations of gases and glucose among the groups in this study (Supplementary Tables 1, 2). Thus, it is unlikely that anesthesia and/or surgery induced neurodegeneration in the hippocampus via hypoxia, hypercapnia or hypoglycemia.

## DISCUSSION

In the present study, we explored the relationship of autophagy to anesthesia/surgery-induced early cognitive dysfunction and α-synuclein oligomerization *in vivo*. Our results suggested that impaired hippocampal autophagy contributed to the early cognitive dysfunction, abnormal α-synuclein oligomer aggregation and altered neurotransmitter levels in the hippocampus following propofol anesthesia and laparotomy in aged rats. The effects of propofol anesthesia with or without surgery seemed to be short-term, since no changes in autophagic protein expression, α-synuclein oligomerization or neurotransmitter levels in the hippocampus were observed 18 weeks after the surgical and anesthetic challenges.

Propofol is an intravenous sedative-hypnotic that is commonly used for general anesthesia and conscious sedation during medical procedures, as well as for prolonged sedation in intensive care units. Mardini et al*.* found that propofol (alone or combined with surgery) had minimal effects on cognitive function in the 3xTgAD Alzheimer’s transgenic mouse model [18]. Other preclinical studies have demonstrated that propofol can mitigate post-anesthetic [19], postoperative [20] or aging-related cognitive changes [21]. In addition, Lee et al*.* found that propofol anesthesia for 2 h in aged rats was not associated with persistent memory effects [22]. However, impairments in learning and memory behavior caused by propofol anesthesia have been documented in a number of reports [23–26]. The reasons for such discrepancies are unclear, but may be due to differences in the experimental designs, animal models or pre-existing vulnerabilities. In our study, propofol anesthesia alone for 4 h but not 2 h caused neurobehavioral deficits in aged rats, suggesting that longer exposures and higher doses of propofol may cause cognitive dysfunction in aged rats. In line with this, a previous study demonstrated that propofol anesthesia alone for 3 h caused spatial memory impairments for up to two weeks after anesthesia in aged rats [27]. Of note, while propofol anesthesia for 2 h alone had no effect in the present study, it induced learning and memory impairments when combined with laparotomy. This indicates that surgical trauma and anesthesia may synergize to induce POCD.

α-Synuclein is primarily located in presynaptic terminals. Thus, to detect α-synuclein expression, we isolated synaptosomes, which are known to contain nerve terminals. While total α-synuclein levels in the hippocampus were not altered, α-synuclein oligomer levels were significantly elevated following 4-h propofol anesthesia or 2-h propofol anesthesia combined with surgery. We also found that anesthesia alone or combined with surgery increased α-synuclein oligomer levels in soluble components, where they are thought to be the most harmful to synaptic and cognitive function [12, 28, 29]. It is reported that oligomeric α-synuclein may induce neurotoxicity by causing mitochondrial defects [30], membrane damage [31], autophagic and lysosomal dysfunction [32], endoplasmic reticulum stress [33], inflammatory responses [34], seeding and spreading [35] or synaptic dysfunction [36]. In fact, our research group has found that α-synuclein oligomers induced by anesthesia and surgery in POCD rats exhibit mitochondrial toxicity by engineered ascorbate peroxidase (APEX) technology (unpublished data). Much more work needs to be done to elucidate the direct relationship between α-synuclein oligomerization and cognitive deficits.

Autophagy is believed to be important for the degradation of α-synuclein aggregates [37]; thus, autophagic and lysosomal dysfunction could lead to the aggregation of α-synuclein and the overproduction of detrimental oligomers. Lee et al. found that α-synuclein oligomers were cleared by a lysosomal mechanism, and that a lysosomal block induced α-synuclein aggregation and toxicity in vitro [32]. Similarly, bafilomycin (an inhibitor of lysosomal and autophagosomal fusion) increased exosomal α-synuclein levels, while rapamycin reversed this effect [38]. It is well known that rapamycin is a specific inhibitor of mTORC1 (the mammalian target of rapamycin complex 1), not a specific agonist of autophagy. The mammalian target of rapamycin (mTOR) signaling pathway is a master regulator of cell growth and metabolism. mTOR exists in two functionally distinct complexes referred to as mTORC1 and mTORC2. mTORC1 integrates multiple signals and modulates crucial cell functions, including cell growth and proliferation, energy metabolism, anabolic and catabolic processes [39]. As a specific inhibitor of mTORC1, rapamycin regulates all of aforementioned processes, including macroautophagy, one of the main intracellular degradation pathways [40]. In addition, our previous and some other studies indicate that rapamycin treatment can ameliorate cognitive dysfunction through improving hippocampal autophagy in POCD rats [5, 7, 41]. We therefore adopted rapamycin as an autophagy agonist in this study.

In the present study, we found that the rapamycin pretreatment reversed the suppressed hippocampal autophagy induced by anesthesia/surgery. At the same time, the pathological accumulation of α-synuclein oligomers was also ameliorated by rapamycin. Thus, the inhibition of autophagy may probably responsible for the hippocampal accumulation of α-synuclein oligomers following anesthesia and anesthesia/surgery. Further studies are necessary to determine the specific molecular mechanisms of the interaction between α-synuclein oligomers and autophagy in this POCD model.

The cornu ammonis 1 (CA1) region is the output of the hippocampus. Pathological damage to the CA1 region causes memory deficits [42, 43]. Moreover, neurogenesis in the dentate gyrus (DG) correlates positively with learning and memory, and its disruption induces memory deficits [44]. We analyzed LC3B expression in these two regions by both immunostaining and Western blotting, and found that propofol anesthesia (4 h) or propofol anesthesia (2 h) in combination with surgery reduced LC3B expression in the CA1 region and the DG. These results demonstrate that 4-h propofol exposure or 2-h propofol exposure combined with surgery inhibited hippocampal autophagy in aged rats. These effects were reversed by pretreatment with rapamycin.

We also found that propofol alone or combined with surgery altered the levels of three neurotransmitters (norepinephrine, dopamine and 5-hydroxytryptamine) in the hippocampus. Imbalanced neurotransmitter levels are thought to be important risk factors for the development of POCD [45]. POCD reduced the levels of the inhibitory neurotransmitter 5-hydroxytryptamine in the rat hippocampus, while rapamycin upregulated this neurotransmitter. All these changes corroborated with behavioral changes. A recent study indicated that 5-hydroxytryptamine inhibited α-synuclein amyloid fiber maturation, and instead promoted and stabilized intermediate aggregates [46]. Future studies are needed to elucidate the possible casual linkages from neurotransmitter imbalances to α-synuclein aggregation to POCD development in response to anesthesia with or without surgery.

Persistent cognitive impairment has been noted in 10% of elderly patients up to three months after surgery [3]. Surprisingly, in aged rats, we did not detect any significant pathological changes (including autophagy inhibition) 18 weeks after anesthesia or anesthesia combined with surgery. The results from animal models may differ from clinical scenarios. In addition, given that POCD does not develop in all patients after surgery, anesthesia- and anesthesia/surgery-induced changes to the brain and other organs may undergo self-limitation and healing, such that relatively limited pathological changes are detectable at later time points.

There are several limitations to the current study. First, not all α-synuclein oligomers are toxic, however, we did not determine the proportions of toxic and nontoxic α-synuclein oligomers. Toxic α-synuclein oligomers bind to the membrane surface more strongly and disrupt the lipid bilayer more severely than nontoxic oligomers in neuronal cells [47]. Second, it is widely known that α-synuclein monomers have the physiological function of regulating the neurotransmitter balance in the brain [11, 48]. So the monomer-to-polymer ratio was also a meaningful indicator for evaluating α-synuclein toxicity. We have found that both propofol anesthesia alone for 4 h and propofol anesthesia for 2 h combined with a laparotomy surgery significantly decreased the ratio of monomer to polymer α-synuclein 7 days after anesthesia/surgery challenges. Moreover, rapamycin pretreatment significantly reversed these changes of monomer-to-polymer ratios. However, we focus on α-synuclein oligomer in the present study and the effect of anesthesia/surgery on monomer and polymer α-synuclein is our next step exploration. Third, no analgesics were used during the surgical procedure, so the contributions of noxious stimuli to the development of POCD are unknown. Fourth, we focused on the hippocampus in this study. Other forebrain regions such as the amygdala and prefrontal cortex have been implicated in cognitive function, but we did not investigate them in this study.

**Figure 5:**
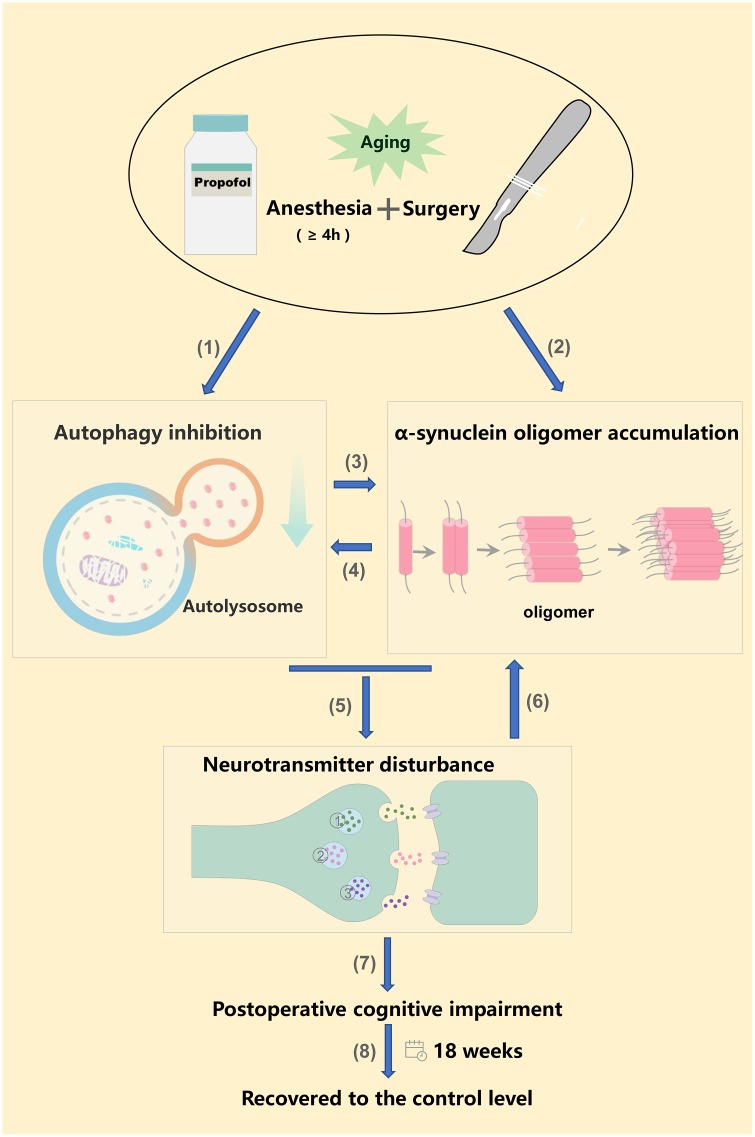
**Anesthesia and surgery inhibited autophagy, increased α-synuclein oligomer levels, ultimately altering neurotransmitter levels and promoting POCD.** (1) (2) Long-term propofol anesthesia (≥ 4 h) or surgery can inhibit autophagy and promote α-synuclein oligomer accumulation in the hippocampus; (3) Hippocampal autophagy inhibition further promotes α-synuclein oligomer accumulation; (4) Hippocampal α-synuclein oligomer accumulation further aggravates the inhibition of autophagy [57, 58]. (5) Hippocampal autophagy inhibition and α-synuclein oligomer accumulation alter neurotransmitter levels, ultimately promoting POCD (7); (6) Neurotransmitter imbalances may further exacerbate α-synuclein oligomer aggregation [46]. (8) Neurobehavior, autophagy-related protein levels and α-synuclein oligomer levels returned to the control levels 18 weeks post-surgery or post-anesthesia. ① Norepinephrine; ② Dopamine; ③ 5-hydroxytryptamine.

Nevertheless, our study demonstrated that anesthesia and surgery increased α-synuclein oligomerization and disturbed neurotransmitter homeostasis in the hippocampus, possibly by locally inhibiting autophagy. These sequential, interrelated pathological events may ultimately promote the development of POCD. If our data can be extrapolated to clinical settings, they may facilitate the development of novel pharmacotherapies to prevent and treat POCD Figure 5.

## MATERIALS AND METHODS

### Animals and ethics

Twenty-month-old male Sprague Dawley rats (550-650 g) were obtained from the Dongchuang Laboratory Animal Center (Changsha, Hunan, China). The temperature (25°C) and humidity (60%) of the animal facility were controlled. The rats were allowed free access to food and water, and were given two weeks to acclimate to the environment before the experiments. The experimental protocol was approved by the Peking University Biomedical Ethics Committee Experimental Animal Ethics Branch (Certification number: LA201413), and followed the National Guidelines of the Administration of Laboratory Animals in China and the Humane Treatment of Laboratory Animals in China.

### Anesthesia and surgery

The rats were injected intraperitoneally with propofol at a dose of 50 mg/kg (Diprivan; Astra-Zeneca, London, UK) to induce anesthesia. After the loss of the righting reflex, the lateral tail vein was cannulated with a 24G intravenous catheter. The rats were infused intravenously with propofol at 0.9 mg/kg/min for 2 h (the PRO2 group) or 4 h (the PRO4 group), as reported previously [7].

Laparotomy was performed aseptically, as described previously [49]. Briefly, a 3-cm vertical incision was made 5 mm below the lower right rib. The researcher’s index finger was inserted up to the second knuckle into the opening, and the viscera and musculature were vigorously manipulated. About 10 cm of the intestine was exteriorized and vigorously rubbed between the surgeon’s thumb and index finger for 30 seconds. The intestine was then placed back into the peritoneal cavity, and the skin was sutured with surgical staples. The surgery lasted 20-25 min. For surgical pain relief, EMLA cream (2.5% lidocaine and 2.5% prilocaine) was applied to the incision wound at the end of the surgery and then every 8 h for two days. Some cohorts with or without propofol/surgery were treated with rapamycin (see below).

### Experimental arrangements and groups

The rats that were administered the vehicle (intralipid) or propofol prior to laparotomy were randomly assigned to the following experiments.

### Experiment A

### Effects of propofol anesthesia alone on neurobehavior

The effects of propofol anesthesia alone on learning and memory behaviors were examined in aged rats. The rats were assigned to three groups (n = 20 per group): control, propofol anesthesia for 2 h and propofol anesthesia for 4 h. The control animals were treated with the vehicle, while the other two groups were anesthetized with propofol and then maintained under anesthesia for 2 or 4 h. The MWM task (n = 10) and the FCT (n = 10) were started to perform on days 1 and 2 post-anesthesia, respectively.

### Effects of propofol anesthesia plus surgery on neurobehavior

The effects of propofol anesthesia plus surgery on learning and memory behaviors were determined in aged rats. The animals were randomly divided into two groups (n = 20 per group): the control group, in which the rats were subjected to vehicle infusion and sham operations, and the anesthesia plus surgery group, in which the rats underwent laparotomies during 2-h propofol anesthesia. Learning and memory behaviors were evaluated with the MWM task and the FCT (n = 10 per test).

### Experiment B

### Effects of anesthesia alone on autophagy flux, α-synuclein expression and α-synuclein oligomerization

The effects of anesthesia alone on autophagy flux, α-synuclein expression and α-synuclein oligomerization in the hippocampus were investigated in aged rats. The grouping design was the same as in experiment A: rats were assigned to the control, 2-h propofol or 4-h propofol group (n = 18 each). On days 1, 3 and 7 after the anesthesia challenge, six rats from each group were randomly selected, and hippocampal samples were obtained. The levels of autophagy-related proteins (LC3B, Beclin-1 and p62), α-synuclein and α-synuclein oligomers were determined by Western blotting.

### Effects of anesthesia plus surgery on autophagy flux, α-synuclein expression and α-synuclein oligomerization

The effects of propofol anesthesia plus surgery on hippocampal autophagy and α-synuclein expression were evaluated in another cohort of rats. Rats were randomly assigned to the control group or the anesthesia/surgery group, as described in experiment A (n = 18 each). The hippocampal levels of autophagy markers, α-synuclein and α-synuclein oligomers were detected by Western blotting.

### Experiment C

### Short- and long-term effects of autophagy activation on α-synuclein oligomerization, neurotransmitter levels and neurobehavior after 4-h propofol anesthesia

To evaluate whether impaired autophagy contributed to propofol-induced cognitive decline, we used the autophagy agonist rapamycin to activate autophagy flux. Rapamycin was intraperitoneally injected at 10 mg/kg once daily for five consecutive days. On the sixth day, rapamycin was intraperitoneally injected (10 mg/kg) 1 h before intralipid or propofol injection. This dosing protocol protected against propofol-induced memory deficits in our previous study [7].

The effects of rapamycin pretreatment on autophagy flux, α-synuclein oligomerization, neurotransmitter levels and neurobehavior were explored within one week or 18 weeks [18] after propofol exposure. Specifically, autophagy flux and α-synuclein oligomerization were assayed by Western blotting 1, 3 and 7 days and 18 weeks post-anesthesia (n = 6 at each time point). Neurotransmitter levels were determined on day 1 and week 18 following propofol treatment (n = 8), while LC3B immunofluorescence was detected on day 7 and week 18 (n = 4) post-anesthesia.

### Experiment D

### Short- and long-term effects of autophagy activation on α-synuclein oligomerization, neurotransmitter levels and neurobehavior after anesthesia plus surgery

We also explored the short- and long-term effects of rapamycin pretreatment on autophagy flux, α-synuclein oligomerization, neurotransmitter levels and neurobehavior after laparotomy under propofol anesthesia. The rapamycin dosing protocol and the experimental design were the same as in Experiment C.

### Blood gas analysis

Blood gas analysis was conducted to determine whether the experimental procedures caused physiological disturbances such as hypoxia, hypercapnia or hypoglycemia. At the end of anesthesia exposure in Experiments C and D, blood samples (0.5 mL) were taken from rats (n = 6 each) via cardiac puncture for arterial blood gas analysis (OPTI Medical Systems, Inc., Roswell, GA, USA) and blood glucose measurement (Life Scan Inc., Milpitas, CA, USA) [50]. These rats were not used for any further analyses.

### MWM

Twenty-four hours after anesthesia or surgery, cognitive function was assessed by the MWM test (Sunny Instruments Co. Ltd., Beijing, China), as previously described [7]. The test was conducted by experimenters who were blinded to the experimental protocols. Swimming was video-tracked (Sunny Instruments Co. Ltd.). The rats’ latency, path length, swimming speed, time and distance to the first platform crossing, and time spent in the target quadrant were analyzed.

### FCT

The FCT (Xeye Fcs; MacroAmbition S&T Development Co. Ltd., Beijing, China) was performed in accordance with a previous study [51] with minor modifications. Briefly, the pairing of context/tone and stimuli was performed 2 h after anesthesia or anesthesia with surgery. The first and second context and tone tests were performed 2 and 7 days after the end of the pairing, respectively. Learning and memory function in the context and tone tests were assessed based on the amount of time that the rats demonstrated “freezing behavior,” defined as a completely immobile posture other than respiratory efforts.

### Extraction of synaptosomes

Synaptosomes were isolated as in previous studies [52, 53]. In short, hippocampal tissue collected on day 1, 3 or 7 after anesthesia or surgery was homogenized and centrifuged at 10,000 × *g* at 4°C for 10 min. The supernatant was centrifuged at 17,000 × *g* at 4°C for 20 min to obtain the P2 fraction for further Percoll (Pharmacia Biotech, Uppsala, Sweden) gradient separation. To maximize the yield of synaptosomes, we collected the fractions between 10% and 23% for Western blotting analysis. The final pellets were centrifuged twice at 17,000 × *g* at 4°C for 20 min, and were resuspended in Tris-HCl buffer (pH 7.4) in accordance with the experimental requirements. For the detection of α-synuclein oligomers, the pellet was resuspended in SDS and centrifuged again. The synaptosomal protein concentration was determined with a bicinchoninic acid assay (Pierce, Rockford, IL, USA).

### Collection of soluble proteins

A graded extraction was performed to biochemically fractionate α-synuclein from the SDS-solubilized hippocampal samples, as described previously [54, 55].

### Western blot analysis

Western blotting was performed with primary (rabbit) antibodies against LC3 (1:1,000; Abcam, Cambridge, MA, USA), Beclin-1 (1:1,000; CST, Danvers, MA, USA), p62 (1:1,000; CST), α-synuclein (1:1,000; Abcam) or β-actin (1:5,000; Applygen, Beijing, China). A fluorescently labeled secondary antibody (1:10,000; LI-COR Biosciences, Lincoln, NE, USA) was used.

### Detection of neurotransmitters

The neurotransmitters norepinephrine, dopamine and 5-hydroxytryptamine were detected in hippocampal synaptosomes by high-performance liquid chromatography with electrochemical detection, as described previously [56]. Briefly, the six-channel detector potentials were set to +50, 100, 200, 300, 400 and 500 mV with a glassy carbon electrode and an Ag/AgCl reference electrode. The mobile phase was delivered to the reversed-phase column at a flow rate of 1 mL/min at 22°C. Ten-microliter aliquots were injected by an auto-injector, and the cooling module was set to 4°C. Data were calculated through external standard calibration.

### Immunofluorescent staining

Immunofluorescent staining was performed to determine the total endogenous levels of LC3B protein, as in our previous study [7]. Both the CA1 and DG regions were analyzed. In brief, we used a primary LC3B antibody (#3868; 1:200; CST) and a secondary Alexa-Fluor 488 antibody (#ab150077; 1:100; Abcam). Nuclei were counterstained with 4’,6-diamidino-2-phenylindole (1:5,000; Roche, Mannheim, Germany). Images were captured with a confocal fluorescence microscope (TCS SP8 X, Leica).

### Statistical analysis

Data are presented as the mean ± standard error of the mean (SEM), and were analyzed with one- or two-way analysis of variance followed by the Bonferroni post-hoc test wherever appropriate (SPSS 16.0 for Windows, IBM, Armonk, NY, USA). A p value less than 0.05 was considered statistically significant.

## Supplementary Material

Supplementary Figures

Supplementary Tables
