# Supplementary material for: Autophagy prevents hippocampal α-synuclein oligomerization and early cognitive dysfunction after anesthesia/surgery in aged rats
Source: Aging (Albany NY). 2020 Apr 26;12(8):7262–81. doi: 10.18632/aging.103074 (PMC7202547; doi:10.18632/aging.103074)
Supplement: Supplementary Figures [file aging-12-103074-s002..pdf]

SUPPLEMENTARY FIGURES

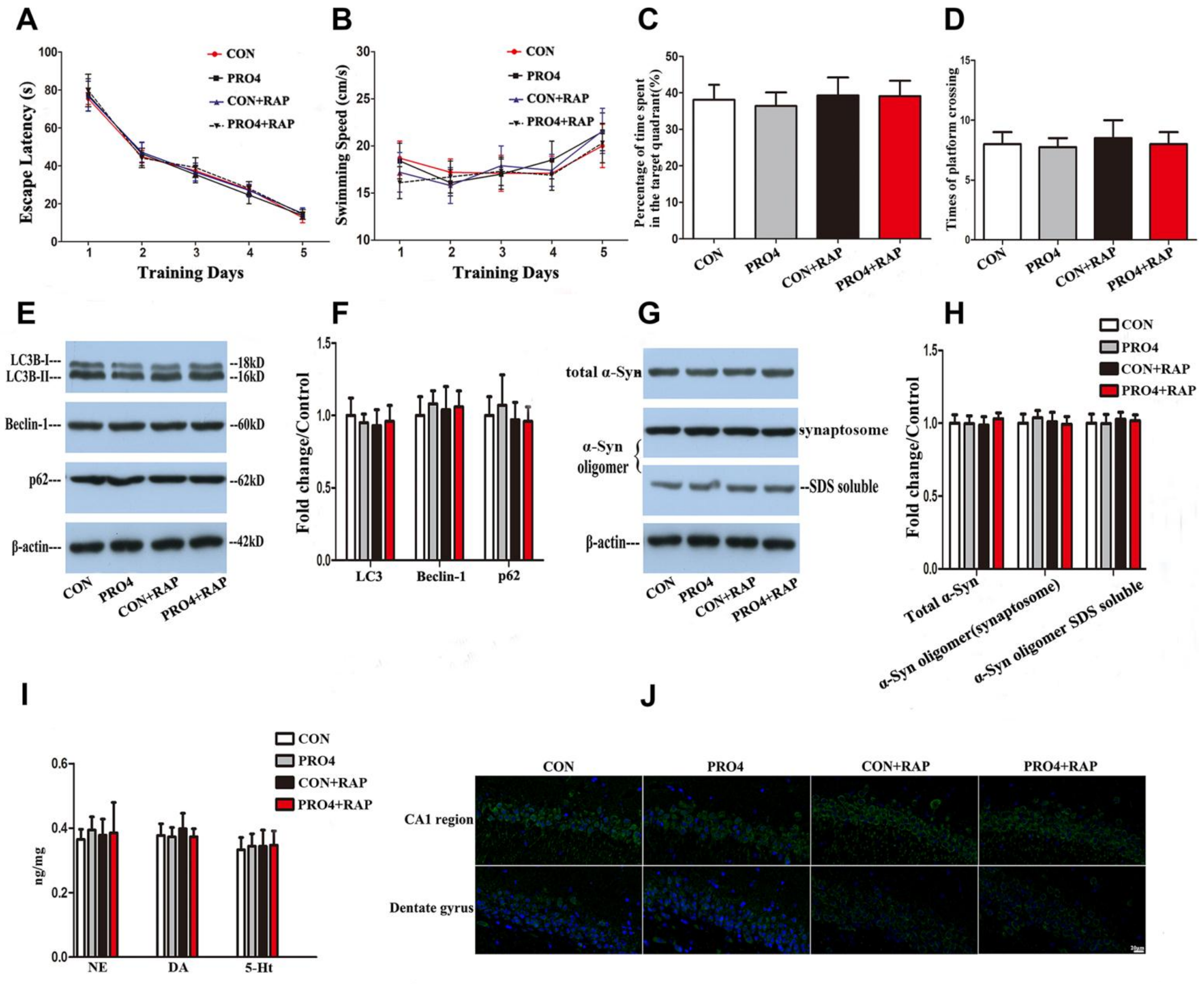

Supplementary Figure 1. Long-term prognosis for neurobehavioral tests and α-synuclein and autophagy-related protein expression in aged rats 18 weeks after propofol anesthesia alone. (**A**–**D**) There were no significant differences in the Morris water maze test results of the PRO4 group compared with the control group 18 weeks after anesthesia alone. (**E**, **F**) Autophagy-related proteins recovered to control levels 18 weeks after anesthesia. (**G**, **H**) α-synuclein oligomers recovered to control levels 18 weeks after anesthesia alone. (**I**) Neurotransmitters recovered to control levels 18 weeks after anesthesia alone. (**J**) There was no significant difference in LC3B expression in the hippocampus 18 weeks after anesthesia alone. n=10 in each group for neurobehavioral tests, n=8 in each group for neurotransmitter detection, n=6 in each group for western blot analysis, and n=4 in each group for confocal of LC3B staining. CON: the control group, CON+RAP: the control+rapamycin group, PRO4: the 4-h propofol anesthesia group, PRO4+RAP: the 4-h propofol anesthesia+rapamycin group. α-Syn: α-synuclein. Scale bar, 20 µm (R).

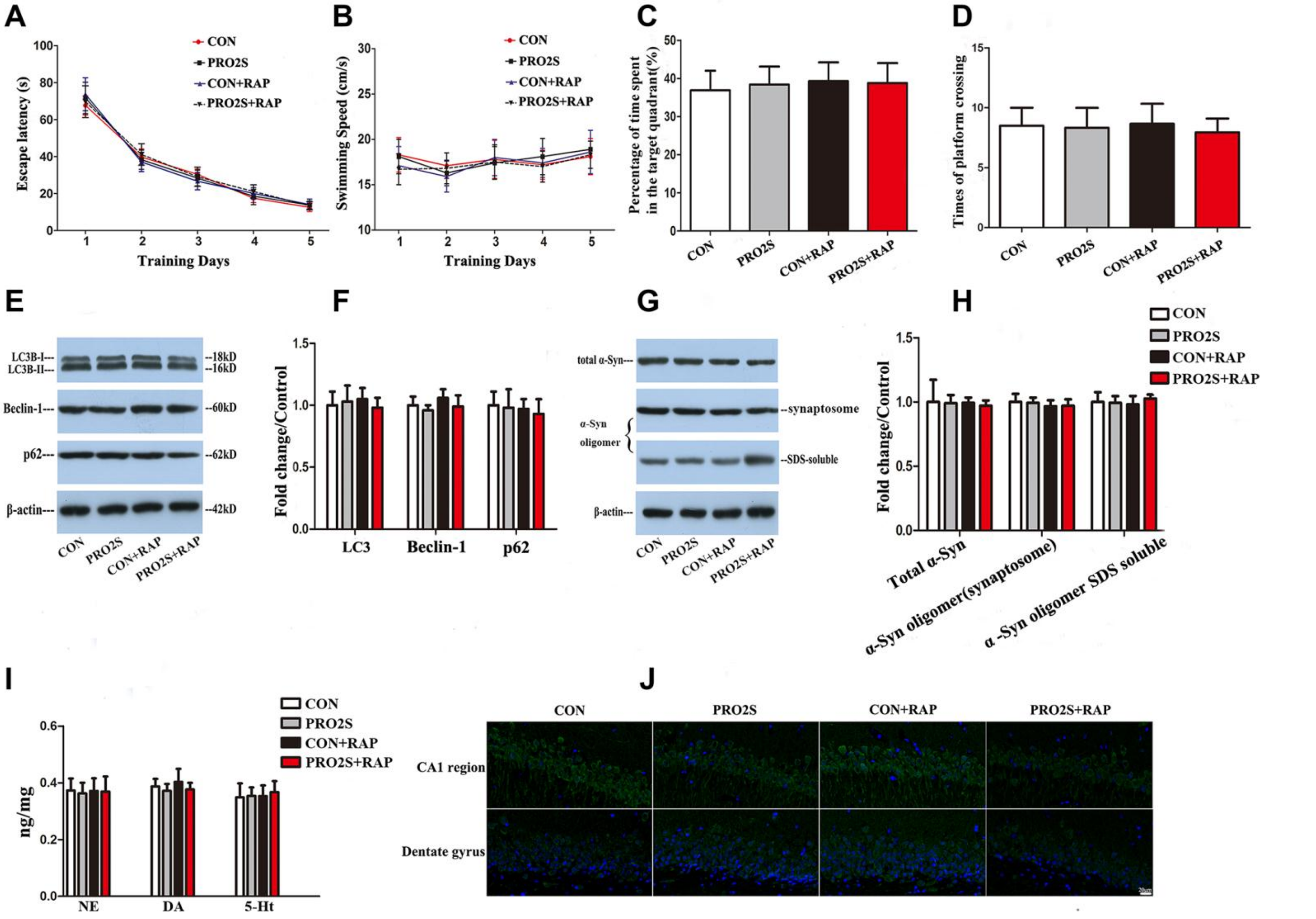

Supplementary Figure 2. Long-term prognosis for neurobehavioral tests and autophagy-related protein and α-synuclein expression in aged rats 18 weeks after anesthesia and surgery. (**A**–**D**) There were no significant differences in the Morris water maze test results of the PRO2S group compared with the control group 18 weeks after surgery. (**E**, **F**) Autophagy-related proteins recovered to control levels 18 weeks after surgery. (**G**, **H**) α-synuclein oligomers recovered to control levels 18 weeks after surgery. (**I**) Neurotransmitters recovered to control levels 18 weeks after surgery. (**J**) There was no significant difference in LC3B expression in the hippocampus 18 weeks after surgery. n=10 in each group for neurobehavioral tests, n=8 in each group for neurotransmitter detection, n=6 in each group for western blot analysis, and n=4 in each group for confocal of LC3B staining. CON: the control group, CON+RAP: the control + rapamycin group, PRO2S: the 2-h propofol anesthesia+surgery group, PRO2S+RAP: the 2-h propofol anesthesia+surgery+rapamycin group. α-Syn: α-synuclein. Scale bar, 20 µm (R).
