# Supplementary material for: Autophagy prevents hippocampal α-synuclein oligomerization and early cognitive dysfunction after anesthesia/surgery in aged rats
Source: Aging (Albany NY). 2020 Apr 26;12(8):7262–81. doi: 10.18632/aging.103074 (PMC7202547; doi:10.18632/aging.103074)
Supplement: Supplementary Tables [file aging-12-103074-s001..pdf]

SUPPLEMENTARY TABLES

Supplementary Table 1. Effects of propofol anesthesia alone on blood gas and blood glucose in aged rats.

Group	n	PH	$PaCO_2$ (mmHg)	PaO_2 (mmHg)	SaO_2 (%)	Hb (g/L)	Glucose (mmol/L)
CON	6	7.35±0.02	41.1±3.3	157.3±14.0	96.7±2.5	151.7±14.5	6.3±0.6
PRO4	6	7.39±0.07	42.9±3.7	153.3± 8.2	95.7±1.6	146.3± 7.7	6.2±0.7
CON+RAP	6	7.37±0.04	41.7±1.4	155.7±14.6	97.0±1.0	151.4±10.0	6.2±0.3
PRO4+RAP	6	7.38±0.02	42.4±3.0	154.7±11.5	95.1±0.69	148.8± 7.9	6.0±0.5
P Value		0.491	0.367	0.270	0.240	0.929	0.850

Propofol intravenous anesthesia for 2 or 4 hours had no significant effect on blood gas and blood glucose in aged rats, and this was not influenced by rapamycin. $PaCO_2$, partial pressure of carbon dioxide in arterial blood; PaO_2, partial pressure of oxygen in arterial blood; SaO_2, arterial oxygen saturation; Hb, hemoglobin; Glucose, blood glucose. CON: the control group, CON+RAP: the control+rapamycin group, PRO4: the 4-h propofol anesthesia group, PRO4+RAP: the 4-h propofol anesthesia+rapamycin group. Values are shown mean± SEM (n = 6/group).

Supplementary Table 2. Effects of propofol anesthesia and surgery on blood gas and blood glucose in aged rats.

Group	n	PH	$PaCO_2$ (mmHg)	PaO_2 (mmHg)	SaO_2 (%)	Hb (g/L)	Glucose (mmol/L)
CON	6	7.34±0.04	42.1±2.7	158.1±13.6	96.1±3.5	149.7±10.5	6.4±0.5
PRO2S	6	7.38±0.06	42.7±3.9	154.7±9.2	95.9±2.3	143.3± 5.7	6.4±0.7
CON+RAP	6	7.35±0.04	42.3±3.0	158.7±10.5	96.9±1.39	149.8± 8.4	6.2±0.7
PRO2S+RAP	6	7.37±0.06	42.7±3.4	155.7±14.6	95.0±1.0	150.4±10.5	6.2±0.4
p value		0.497	0.387	0.283	0.276	0.892	0.861

Intravenous propofol anesthesia for 2 hours combined with laparotomy had no significant effect on blood gas and blood glucose in aged rats, and this was not influenced by rapamycin. $PaCO_2$, partial pressure of carbon dioxide in arterial blood; PaO_2, partial pressure of oxygen in arterial blood; SaO_2, arterial oxygen saturation; Hb, hemoglobin; Glucose, blood glucose. CON: the control group, CON + RAP: the control + rapamycin group, PRO2S: the 2-h propofol anesthesia+surgery group, PRO2S+RAP: the 2-h propofol anesthesia+surgery+rapamycin group. Values are shown mean± SEM (n = 6/group).
